# Advancements in Antiviral Drug Development: Comprehensive Insights into Design Strategies and Mechanisms Targeting Key Viral Proteins

**DOI:** 10.4014/jmb.2403.03008

**Published:** 2024-04-29

**Authors:** Wang Hangyu, Li Panpan, Shen Jie, Wang Hongyan, Wei Linmiao, Han Kangning, Shi Yichen, Wang Shuai, Wang Cheng

**Affiliations:** 1 Key Laboratory of Immune Microenvironment and Inflammatory Disease Research in Universities of Shandong Province, School of Basic Medical Sciences, Shandong Second Medical University, Weifang 261053, P.R. China; 2 School of Medical Laboratory, Shandong Second Medical University, Weifang 261053, P.R. China; 3 School of Stomatology, Shandong Second Medical University, Weifang 261053, P.R. China; 4 Department of Rheumatology and Immunology, The Affiliated Hospital of Inner Mongolia Medical University, Inner Mongolia 010050, P.R. China; 5 Inner Mongolia Key Laboratory for Pathogenesis and Diagnosis of Rheumatic and Autoimmune Diseases, Inner Mongolia 010110, P.R. China

**Keywords:** Antiviral drugs, virus-targeted drugs, viral life cycle, drug development, targets of antiviral drugs

## Abstract

Viral infectious diseases have always been a threat to human survival and quality of life, impeding the stability and progress of human society. As such, researchers have persistently focused on developing highly efficient, low-toxicity antiviral drugs, whether for acute or chronic infectious diseases. This article presents a comprehensive review of the design concepts behind virus-targeted drugs, examined through the lens of antiviral drug mechanisms. The intention is to provide a reference for the development of new, virus-targeted antiviral drugs and guide their clinical usage.

## Introduction

Throughout human history, viruses related to pestilence, such as variola virus, influenza virus, Middle East respiratory syndrome coronavirus (MERS-CoV), and severe acute respiratory syndrome coronavirus 2 (SARS-CoV-2), have wreaked havoc in many countries. In particular, the global SARS-CoV-2 pandemic has comprehensively impacted public health while causing serious socio-economic repercussions worldwide. However, such highly infectious virus-associated diseases have few effective drugs, and furthermore, rapid viral mutation and drug resistance have placed greater demands on the development of new antiviral drugs [1-4].

The life cycle of viruses consists of adsorption, penetration, uncoating, biosynthesis, assembly maturation and release, and each step is critical to the formation of intact virions that can infect their host [5]. Intrinsically, hindering or disturbing any process of the viral life cycle enables the achievement of antiviral effects. Here, we present a comprehensive understanding of the shared characteristics, targets, and mechanisms of action of existing virus-targeted drugs, approached from the standpoint of antiviral drug mechanisms. We specifically focus on respiratory viruses, hepatitis viruses, and retroviruses as examples, aiming to provide insights for the development of new antiviral drug mechanisms.

## Antiviral Drug Development Strategies

The development of antiviral drugs typically falls into two major categories. The first approach employs broad-spectrum antiviral strategies, such as utilizing Type I interferons to target the host cell Janus kinase-signal transducer and activator of transcription (JAK-STAT) pathway. This promotes the expression of various antiviral proteins such as interferon-stimulated genes (ISGs) in the host cells to inhibit viral infection. Alternatively, a more targeted approach can be taken that directly interferes with critical stages in the viral life cycle to inhibit viral replication. This involves the use of drugs that combine and inhibit the viral polymerases, proteases, and capsid proteins introduced in this paper. Antiviral drug development may either repurpose existing medications or explore novel compounds. Theoretically, targeting pivotal proteins or intrinsic nucleic acids involved in the viral replication cycle can effectively inhibit viral replication [6].

## Key Molecule Targets of the Viral Life Cycle

**Fig. 1:**
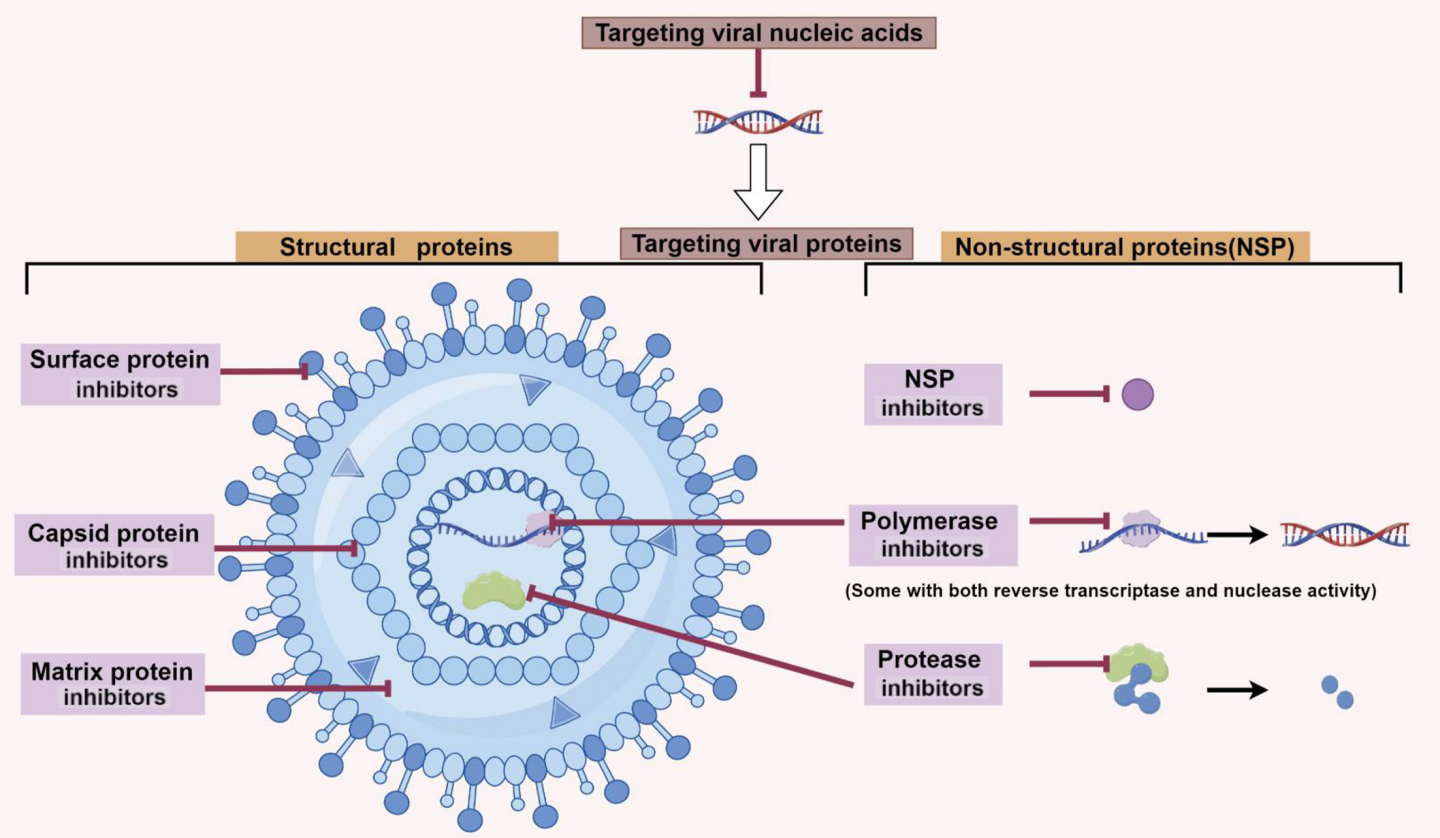
Design strategies for virus-targeted antiviral drugs (by Figdraw).

Virus-targeted antiviral drugs, also known as virus-targeted drugs, aim to interfere with the normal life cycle of a virus by targeting key proteins or viral nucleic acids involved in the viral life cycle. Essentially, all key viral proteins can serve as targets for antiviral drugs. These include viral structural proteins and viral non-structured proteins (NSP) which have important biological functions [7-10]. In addition, inhibition of viral nucleic acids can also directly block viral replication [11, 12]. Currently, virus-targeted drugs predominantly adopt a focused targeted strategy, primarily targeting viral polymerases, proteases, capsid proteins, and other protein targets (Fig. 1, Tables 1-5).

## Targeting Viral Surface Proteins

The exterior viral protein, prominently displayed on the outermost periphery of the virion, plays a quintessential role in mediating both adsorption and subsequent penetration of the virus into host cells. This protein constitutes an imperative target for vaccine development, immunoglobulin synthesis, and antiviral pharmacotherapy. The initial processes of adsorption and penetration serve as the inaugural stages in the cascade of viral infection. Theoretically, impeding these initial viral incursions has the potential to preemptively quash the infectious process at its nascent stage [13]. Influenza viruses have two types of spikes embedded in the envelope, hemagglutinin (HA) and neuraminidase (NA) spikes. These two spike proteins are the most important surface proteins of influenza viruses and are important targets for anti-influenza drugs. Influenza viruses bind to sialic acid (SA) receptors on the surface of host respiratory mucosal epithelial cells through HA and invade the host cells in a cytosolic manner [14, 15]. Currently, the main influenza HA inhibitors identified are Abidor and the polypeptide drug, Flufirvitide-3 [14, 16, 17]. In addition, SA derivatives can also block the specific binding of HA to SA [18, 19]. HA is also the main antigenic structure of the influenza virus, which is the binding site of neutralizing antibodies (nAbs) [20, 21]. NA is a sialidase that promotes viral budding release by hydrolyzing N-acetylneuraminic acid at the end of glycoproteins on the host cell surface [14, 22, 23]. Oseltamivir, Zanamivir and Laninamivir are antiviral drugs that target the NA of influenza viruses [14]. These NA inhibitors are widely used clinically as anti-influenza drugs, but drug resistance is now a common problem [14, 24-26]. SARS-CoV-2 invades host cells by recognizing angiotensin converting enzyme 2 (ACE2) and type II serine proteases (TMPRSS2) on the surface of the host cell through S protein [27-30]. The S protein is one of the important targets for anti-SARS-CoV-2 drugs. Abidor is able to bind S proteins to block their trimerization and thus inhibit SARS-CoV-2 invasion into host cells [31, 32]. nAbs were also used to inhibit SARS-CoV-2 infection by blocking the binding of S proteins to host cell surface receptors [33-35].

The surface glycoprotein gp120 of human immunodeficiency virus type 1 (HIV-1) engages in a covalent interaction with its transmembrane counterpart, glycoprotein gp41, a relationship pivotal to the viral phagocytic proclivities. Initially, gp120 selectively attaches itself to the CD4 receptor, ubiquitously displayed on the surface of the prospective host cells. This initial engagement is succeeded by an association with chemokine co-receptors—either CCR5 or CXCR4—thereby precipitating the disassociation of gp120 from gp41. After this separation, gp41 undergoes a conformational metamorphosis, facilitating the fusion of the viral envelope with the membrane of the targeted host cells [7, 36, 37]. Consequently, therapeutic strategies that precisely target the HIV-1 glycoprotein complexes and abrogate their affinity for host receptors offer a viable pathway for inhibiting viral invasion. Fostemsavir functions as an HIV-1 adsorption inhibitor, primarily targeting gp120 [38]. On a parallel note, enfuvirtide T20, a polypeptide-based pharmaceutical, operates as a fusion inhibitor by specifically interacting with gp41, thereby precluding the amalgamation of the viral and host cellular membranes [39, 40].

## Targeting Viral Capsid Proteins

The capsid proteins in viruses serve as critical architectural constituents, enveloping the intracellular genetic material and safeguarding it against enzymatic degradation by nucleases. Beyond their protective function, these viral capsid proteins are instrumental in orchestrating a complex array of virus-host interactions, and they modulate both viral infectivity and subsequent maturation. Consequently, pharmacological agents designed to target these capsid proteins possess the capability to perturb the viral life cycle, either by compromising capsid integrity or by inhibiting the assembly of capsid protein complexes [8].

The HIV-1 capsid protein, also known as the p24 protein, is composed of the N-terminal domain (NTD) and C-terminal domain (CTD). The action sites of HIV-1 capsid protein inhibitors include CTD, NTD, and CTD-NTD binding sites [41]. Lenacapavir, the first drug to clinically target the HIV-1 capsid protein, is clinically effective in the context of HIV-1 multi-drug resistance [8, 42]. In addition, a number of small molecule inhibitors and peptide inhibitors targeting the p24 protein are in development or undergoing clinical trials [43].

The capsid proteins of the hepatitis B virus (HBV) also stand as crucial pharmacological targets in the development of antiviral therapeutics. Contemporary antiviral agents engineered to interfere with HBV's capsid proteins operate primarily by obstructing the assembly of the HBV capsid complex. These inhibitors encompass a diverse array of molecular classes, notably including dihydropyrimidine inhibitors, benzenesulfonamide inhibitors, and acrylamide inhibitors, among others. Such pharmacological agents hold significant promise for future research endeavors and could potentially expand the therapeutic arsenal against HBV infection [44-47].

## Targeting Viral Polymerase

Viral polymerases constitute a class of enzymes that orchestrate the biosynthesis of viral genomic material and are, notably, conserved across various viral taxa. This enzymatic conservation renders them salient targets in the conceptualization and development of antiviral pharmaceuticals. Depending upon the nature of the viral nucleic acid and the modality of replication, these viral polymerases are taxonomically segregated into DNA-dependent DNA polymerases, RNA-dependent DNA polymerases, and RNA-dependent RNA polymerases (RdRp). Certain viruses, such as HIV, possess polymerases endowed with dual enzymatic functionalities: reverse transcriptase (RT) and nuclease activities.

RdRp of influenza virus consists of three heterologous subunits: polymerase acidic (PA), polymerase base 1 (PB1), and polymerase base 2 (PB2) [48-50]. Various polymerase inhibitors have been engineered to target these distinct subunits: Baloxavir, a PA-specific inhibitor, is efficacious against both influenza A and B viral strains [49, 50]; nucleoside analogues such as ribavirin and favipiravir act as PB1 antagonists [51-53], while pimodivir operates as a PB2-specific inhibitor [54-56]. The SARS-CoV-2 polymerase complex consists of the non-structural proteins (NSPs) NSP12, NSP7, and NSP8 [57]. NSP12 serves as the RdRp component of the SARS-CoV-2 complex, and its crystallographic analysis furnishes a structural framework for the development of RdRp-targeting antiviral agents [57-59]. Among these, nucleoside analogues like remdesivir and favipiravir specifically target the RdRp of SARS-CoV-2 [27, 60-62]. Non-nucleoside analogues such as suramin also bind specifically to the active site of RdRp and have a higher ability to inhibit RdRp activity than remdesivir in vitro [27, 63].

The RT of HIV has an RNA/DNA-dependent DNA polymerase activity. The first anti-HIV drug, suramin, acts on the viral RT to inhibit the replication cycle of the virus [64]. At present, non-nucleoside drugs represented by etravirine and its derivatives are the main HIV polymerase inhibitors in clinical application [65, 66].

HBV polymerase is a DNA polymerase endowed with both RT and RNase H enzymatic activities. Pharmacological agents designed to inhibit this complex enzyme include lamivudine, entecavir, and telbivudine, among others. These drugs function as competitive antagonists of HBV DNA polymerase, thereby obstructing the viral replication process and offering a therapeutic avenue for mitigating HBV infection [67]. The RdRp of HCV, also known as NS5B, is a NSP that plays an important role in the process of viral replication and is an ideal antiviral drug target. The nucleoside analogue, sofosbuvir, is a marketed polymerase inhibitor of NS5B [68, 69].

## Targeting Viral Proteases

Viral proteases represent a cadre of enzymes encoded by viral genomes, responsible for the hydrolytic cleavage of proteinaceous peptide bonds. These enzymes serve to shear and modify precursor proteins, culminating in the generation of mature, functionally active protein variants. Occupying a pivotal role in the viral life cycle, proteases stand as integral targets in the conceptual framework for the advancement of antiviral therapeutics. These enzymes, by virtue of their essential role in viral maturation and replication, provide a compelling locus for pharmacological intervention, thereby offering promising avenues for the development of efficacious antiviral agents.

The asparagine protease of HIV catalyzes the hydrolytic cleavage of the virus's immature polypeptide chains, thereby transforming precursor proteins into discrete functional and structural entities requisite for the assembly of infectious viral particles. Presently, the majority of HIV protease inhibitors employed in the clinical management of HIV infections are peptidomimetics, exemplified by agents like darunavir. These therapeutic compounds obstruct the maturation of viral particles by specifically inhibiting the enzymatic activity of the HIV asparagine protease, thereby offering a robust strategy for impeding the viral replication cycle [70-72].

**Fig. 2:**
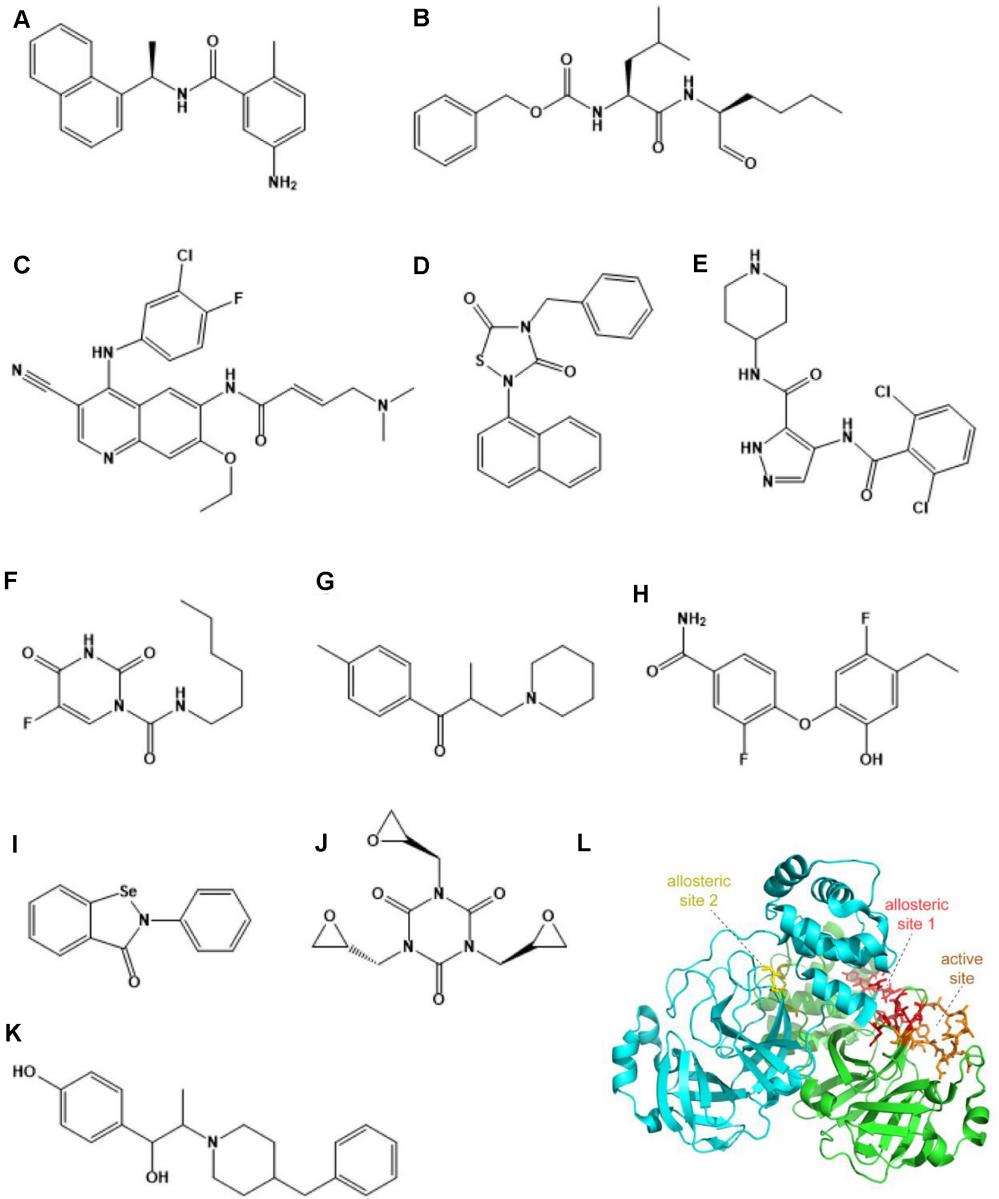
Strategies for targeting the protease of SARS-CoV-2. (**A**) GRL0617. (**B**) Calpeptin. (**C**) Pelitinib. (**D**) Tideglusib. (**E**) AT7519 (**F**) Carmofur. (**G**) Tolperisone. (**H**) MUT056399. (**I**) Ebselen. (**J**) Triglycidyl isocyanurate. (**K**) Ifenprodil. (**L**) A schematic of the main protease of SARS-CoV-2. The active site and allosteric sites are highlighted.

The main protease of coronaviruses is structurally conserved and plays a key regulatory role throughout viral replication, transcription, and assembly, and there is no homologous protease in the human body. Therefore, drugs that target the main protease of coronaviruses may have both reduced toxicity and similar inhibitory effects on multiple coronaviruses. The main protease of SARS-CoV-2, M^pro^, cleaves replicase polyproteins to release NSPs and is an important target for anti-SARS-CoV-2 drug design [27, 73]. Multiple research teams analyzed the structure of M^pro^ and screened some inhibitors (Ebselen, Tideglusib, Carmofur, etc.) targeting M^pro^ as potential anti-SARS-CoV-2 drugs based on its structural design and synthesis. They then reported the crystal structures of M^pro^ and its inhibitors [74-76] (Fig. 2). Furthermore, the reported X-ray structural data demonstrated that α-ketoamides can be used as inhibitors of M^pro^ [77]. Based on the active site of M^pro^ through screening, Pelitinib and other non-toxic compounds have been found with antiviral activity at the cellular level [78]. Lopinavir/ritonavir acting on HIV protease has also shown inhibition of SARS-CoV-2 in vitro [61]. However, clinical trials have confirmed that lopinavir/ritonavir is not effective in vivo [79]. Another protease of SARS-CoV-2, PL^pro^, plays an important role in viral replication and inhibition of host immune response, so designing drugs targeting PL^pro^ is also one of the strategies for drug development [27]. The researchers analyzed the structure of PL^pro^ and found that two peptide-like inhibitors, VIR250 and VIR251, as well as small molecular compounds such as GRL0617, can also bind and inhibit PL^pro^ activity [80-82] (Fig. 2).

Hepatitis virus proteases are not conserved enough, but HCV NS3/4A protein has become an anti-HCV drug target [83, 84]. The NS3/4A protein of HCV is a dimer of cofactor NS4A and NS3 serine protease bound in a non-covalent bond that catalyzes the hydrolysis of the multimeric protein precursor. Grazoprevir and its derivatives are anti-HCV drugs targeting NS3/4A protease [83, 84].

## Targeting Other Structural Proteins of Viruses

In addition to conventional targets like viral polymerases, proteases, and capsid proteins, matrix proteins have also emerged as viable candidates for antiviral drug development. The M2 matrix protein of the influenza virus, an ion channel transmembrane protein, not only constitutes a structural component of the influenza virus but also plays a crucial role in viral replication. Amantadine, the inaugural pharmaceutical agent used against the influenza virus, operates as an M2 matrix protein inhibitor [85]. Amantadine is no longer used for clinical anti-influenza virus therapy due to the emergence of drug resistance, but novel inhibitors designed to target M2 still have the potential to become anti-influenza virus drugs [86].

## Targeting Other Non-Structural Proteins (NSPs) of Viruses

Viruses synthesize NSPs, which are absent from the mature viral particle but remain integral to its life cycle. Noteworthy among these are viral proteases and polymerases, exemplified by the NS3/NS4A protease and NS5B polymerase of the HCV. Additionally, certain NSPs like NS1 and NS2 in the influenza virus, while not categorized as proteases or polymerases, are nonetheless pivotal in the maturation of viral particles and present valuable targets for novel antiviral therapeutics [87]. Epigallocatechine gallate (EGCG) was discovered by high-throughput screening and interfered with the interaction of NS1 with dsRNA, thereby inhibiting influenza [88]. Other NSPs of HCV, notably NS2, serve not only as anti-HCV drug targets but are also anticipated to be incorporated into clinical therapies against HCV [89].

## New Strategies for Targeting Viral Nucleic Acids

Pharmaceutical agents explicitly engineered to target viruses predominantly aim at the virus's essential functional proteins. However, the viral composition is not solely comprised of proteins; it also incorporates nucleic acids. Thus, direct interference with viral nucleic acids may represent a potent antiviral strategy. RNA interference (RNAi) is a ubiquitous, post-transcriptional gene-silencing pathway in eukaryotic organisms, functioning as an efficacious natural defense mechanism to degrade viral RNA within infected cells [90]. The clustered regularly interspaced short palindromic repeats (CRISPR)/CRISPR-associated protein (Cas) gene editing system is an adaptive immune system that has evolved in bacteria and archaea to degrade invading viral or plasmid DNA. The CRISPR/Cas13d system has been used to degrade HIV-1 transcripts and successfully inhibit HIV-1 replication in cell lines [12]. Several studies have demonstrated the ability of the CRISPR/Cas9 system to directly target the HIV genome for anti-HIV effects [11, 91]. Moreover, the CRISPR/Cas9 system has demonstrated considerable efficacy in inhibiting other viruses, including HBV and HCV, thus exhibiting significant therapeutic potential [11].

## Summary and Outlook

Antiviral therapeutics are formulated to strike an optimal balance between potency and safety profiles. Compounds manifesting robust antiviral activity concomitant with minimal adverse effects are logically prioritized for drug development. Monospecific antivirals that demonstrate targeted efficacy can mitigate potential toxicity, while broad-spectrum antivirals serve to obstruct the life cycles of diverse viral entities, thereby extending the drug's clinical applicability. The overarching research paradigm should encompass both these broad-spectrum agents and highly targeted therapies for specific viral entities.

Therapeutics may either target the viral entity directly or focus on host-cell interactions. From the virological standpoint, key proteins integral to the viral life cycle often serve as primary drug targets. Yet, these virus-targeted agents possess inherent limitations, such as ineffectiveness against non-replicating, latently-infected viruses and a susceptibility to resistance development in viruses with high mutational frequencies. Drug resistance is a challenge for clinical treatment, mainly due to the fact that viral nucleic acids are prone to mutation [2, 92]. The nucleic acid sequence changes lead to the structural transformation of the translated proteins that serve as drug targets, such as the viral polymerase, protease, and surface proteins, which in turn cause the drug to be ineffective or show reduced efficacy. To prevent the emergence of drug-resistant mutations, the optimal combination of drug regimen and dosage should be adopted to maximally inhibit viral replication to reduce or delay the emergence of drug resistance [93]. Drug combinations can yield superior clinical outcomes with lower drug resistance, as the treatment of AIDS has adopted highly active anti-retroviral therapy (HAART). Target-based approaches for the design of antiviral drugs can play a pivotal role in combating drug-resistant challenges by enhancing compound-target affinity while minimizing interactions with mutated binding pockets [93]. Viral load monitoring and viral genotyping are carried out to select more appropriate drugs [94-96]. More importantly, new antiviral drugs are being developed to avoid the emergence of multi-drug resistance.

Presently, virus-targeted antiviral drugs do not eradicate the virus but merely hinder its life cycle. However, the host cell not only serves as the substrate for viral parasitism but can also mount an active immune response capable of viral eradication. Therefore, pivotal host-cell molecules and structures may also present viable drug targets [97, 98]. Inhibiting viral replication and facilitating prompt viral clearance from a host perspective remain crucial facets of antiviral therapeutic strategies. Additionally, host-targeting antiviral drugs offer a promising avenue for circumventing viral mutation [93]. In our research we found that RNA modification m^6^A-mediated downregulation of the OGDH-Itaconate pathway reprograms cellular metabolism to inhibit viral replication, proposing potential targets for controlling viral infection [99]. Also, FBL and its mediated RNA Nm modifications inhibit innate immune response, which can promote virus entry into macrophages to facilitate viral infection [100]. So, key regulatory molecules of the host cell could be potential antiviral drug targets with less drug resistance.

Identifying novel pharmacologically active structures and converting them into targeted antiviral agents represents a significant research avenue. Herbal remedies constitute a viable source of antiviral agents [101]. For instance, Shizukaol B, extracted from *Chloranthus japonicus*, displays inhibitory activity against HIV RT [102]. We also found that Chinese herbal monomers can be used as lead compounds to design antiviral drug candidates with better activity and lower toxicity [103, 104]. Moreover, viral RNA cap 2'-O-methyltransferases are considered promising therapeutic targets for antiviral treatments, as they play a key role in the formation of viral RNA cap-1 structures to escape the host immune system, so inhibitors of viral RNA cap 2'-O-methyltransferases present new options [105].

Presently, advanced high-throughput drug screening techniques, including computational virtual screening, DNA-encoded small-molecule libraries, and fluorescence-mediated high-throughput assays, have substantially accelerated the discovery pace of potential antiviral therapeutics [102, 106-109]. New drugs combined with new techniques may lead to major breakthroughs. As the development of novel antiviral agents persists and our understanding of viral pathogenesis deepens, therapeutic strategies for managing viral infections will increasingly diversify.

## Figures and Tables

**Table 1:** Drug targets of viral surface proteins.

Viral target	Drugs or therapy methods
Influenza virus HA inhibitors	Abidor, Flufirvitide-3, SA derivatives, neutralizing antibodies of HA, etc.
Influenza virus NA inhibitors	Oseltamivir, Zanamivir, Laninamivir, etc.
SARS-CoV-2 S protein inhibitors	Abidor, neutralizing antibodies of S protein, etc.
HIV-1 gp120 or gp41 inhibitors	Fostemsavir, enfuvirtide T20, etc.

**Table 2:** Drug targets of viral capsid and matrix proteins.

Viral target	Drugs or therapy methods
HIV-1 p24 inhibitors	Lenacapavir, etc.
HBV Capsid protein inhibitors	Dihydropyrimidine inhibitors, Benzenesulfonamide inhibitors, etc.
Influenza virus M2 inhibitors	Amantadine, Rimantadine, etc.

**Table 3:** Drug targets of viral polymerase.

Viral target	Drugs or therapy methods
Influenza virus RdRp inhibitors	Baloxavir, Ribavirin, Favipiravir, Pimodivir, etc.
SARS-CoV-2 RdRp inhibitors	Remdesivir, Favipiravir, etc.
HIV RT inhibitors	Suramin, Etravirine and its derivatives, etc.
HBV polymerase inhibitors	Lamivudine, Entecavir, Telbivudine, etc.
HCV RdRp inhibitors	Sofosbuvir, etc.

**Table 4:** Drug targets of viral protease and non-structural proteins.

Viral target	Drugs or therapy methods
HIV Protease inhibitors	Darunavir, Lopinavir, Ritonavir, etc.
SARS-CoV-2 M^pro^ inhibitors	Ebselen, Tideglusib, Carmofur, α-ketoamides, Pelitinib, etc.
SARS-CoV-2 PL^pro^ inhibitors	VIR250, VIR251, GRL0617, etc.
HCV NS3/4A protease	Grazoprevir, etc.
HCV NS1 inhibitors	Epigallocatechine gallate, etc.

**Table 5:** Drugs target for viral genome.

Viral target	Drugs or therapy methods
HIV genome	CRISPR/Cas13d system, CRISPR/Cas9 system
HBV genome	CRISPR/Cas9 system
HCV genome	CRISPR/Cas9 system
